# Revisiting the risks of MRI with Gadolinium based contrast agents—review of literature and guidelines

**DOI:** 10.1007/s13244-015-0420-2

**Published:** 2015-08-08

**Authors:** Aurang Z. Khawaja, Deirdre B. Cassidy, Julien Al Shakarchi, Damian G. McGrogan, Nicholas G. Inston, Robert G. Jones

**Affiliations:** Department of Renal Transplant Surgery & Vascular Access, Queen Elizabeth Hospital, University Hospitals Birmingham, Mindelsohn Way, Birmingham, B15 2GW West Midlands UK; Division of Diabetes and Cardiovascular Medicine, University of Dundee, Dundee, DD19SY UK; Department of Radiology, Queen Elizabeth Hospital, University Hospitals Birmingham, Mindelsohn Way, Birmingham, B15 2GW West Midlands UK

**Keywords:** Magnetic resonance imaging, Contrast media, Gadolinium/adverse effects, Nephrogenic fibrosing dermopathy, Renal insufficiency

## Abstract

**Abstract:**

Gadolinium based contrast agents (GBCA) have been linked to the occurrence of nephrogenic systemic fibrosis (NSF) in renal impaired patients. The exact interaction between the various different available formulations and occurrence of NSF is not completely understood, but has been postulated. This association has triggered public health advisory bodies to issue guidelines and best practice recommendations on its use. As a result, the reported incidence of NSF, as well as the published use of GBCA-enhanced magnetic resonance imaging in renal impairment, has seen a decline. Understanding of the events that led to these recommendations can increase clinical awareness and the implications of their usage. We present a review of published literature and a brief overview of practice recommendations, guidelines and manuals on contrast safety to aide everyday imaging practice.

***Teaching Points*:**

• *Low risk gadolinium based contrast agents should be the choice in renal insufficiency*.

• *Higher doses have been linked to NSF development. Doses should be as low as possible*.

• *Clear documentation of date,dose and type of formulation used should be noted*.

• *Post-scan dialysis should be arranged as soon as possible and feasible*.

• *Pre-existing inflammatory state is a risk factor;liver insufficiency is not a contraindication*.

## Introduction

Gadolinium-based contrast agents (GBCA) have been linked to the occurrence of nephrogenic systemic fibrosis (NSF) in renal impaired patients. The majority of studies that report on their use in the renal impaired population were published prior to the publications that prompted the alert on NSF [1–3]. This association has triggered public health advisory bodies to issue guidelines and best practice recommendations on its use in renal insufficiency. Since then, this has all but halted the rapid progression and uptake of contrast-enhanced magnetic resonance imaging (MRI) in this population that was seen in the early to mid 2000s. Understanding of the events that led to these recommendations can increase clinical awareness and the implications of the use of GBCAs in daily imaging practice. We conducted an electronic database search [PubMed/Medline, EMBASE] to collate the evidence in published literature on the occurance of NSF in the renal impaired. We also carried out a forward citation and bibliographic search of identified studies. Published studies were reviewed for reported pathophysiological and clinical manifestations, proposed diagnostic pathway, treatments options and reported incidence. We also reviewed practice recommendations, guidelines and published manuals on contrast safety.

### Background and incidence

In the year 2000, 15 patients with chronic kidney disease were identified presenting with scleromyxoedema-like cutaneous manifestations yet having significant clinical and histo-pathological differences; the term nephrogenic fibrosing dermopathy was initially proposed. These clinical findings are now recognized as characteristic of NSF [4]. Following this, case reports of similar findings and also significant systemic involvement found on autopsy were reported [5, 6]. Patients with end-stage renal disease were reported to develop symptoms as early as two to four weeks after exposure to GBCAs for MRI [1]. Exact pathogenesis remains unclear; however, postulation of likely early dermal manifestation of this gadolinium toxicity is proposed [7]. A strong association is observed in the presence of both acute renal impairment and chronic dialysis dependent renal insufficiency and other influencing co-factors that may play a role, such as a background inflammatory process [8, 9]. As the evidence in published literature increased, the United States Food and Drug Administration (FDA), followed by the European Medicines Agency (EMA) issued an alert on the use of GBCAs in patients with renal insufficiency [10, 11]. Since then, public health and practice guideline bodies have published recommendations on its use [12–14]. A 2008 multi-centre retrospective review reported 15 cases of NSF in a total population of 83,121 (0.02 % incidence), all of whom received at least one administration of a GBCA. All 15 of these cases of NSF were found in patients who had received a higher than standard dose, increasing the incidence to 0.17 % (15 of 8997 patients). A higher than normal dose was described as approximately between 0.2 to 0.4 mmol per kilogram body weight. In the entire cohort, 265 patients were on haemodialysis, but only one of them was reported to have developed NSF (incidence 0.4 %) [3]. Another publication retrospectively collating data from four centres set to determine the benchmark incidence of NSF related to the confirmed use of two GBCAs [15]. They reported an overall incidence of 0.04 % at two centres that used Gadodiamide (32 cases in 82,260 patients—administered total dose range 1 to 9.5 mmol), as compared to the 0.02 % from the previously publish study. The other two centres that used Gadopentate dimeglumin reported an incidence of 0.003 % (four cases in 135,347 patients) with an administered dose ranging between 2.5 and 8.5.

### Clinical findings

**Fig. 1:**
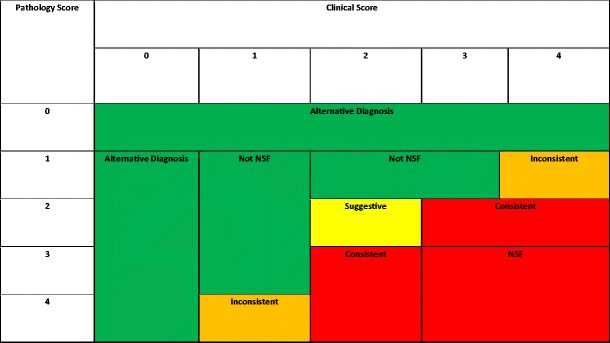
The Girardi Score using clinical criteria and histological findings for diagnosis of NSF [56]

As reported in the literature, specific cutaneous findings on clinical examination with relevant past history of GBCA exposure trigger a differential of NSF, but require histological confirmation [16–18]. It has been postulated that the deposition of disassociated free gadolinium causes this fibrous connective tissue formation [5]. Patients may present with firm, erythematous and indurated plaques of skin associated with subcutaneous oedema. The presentation may range from hyperpigmentation, yellow papules or plaques, blistering or even ulceration [1, 2, 8]. Resultant manifestations include pain, severe pruritus, paraesthesia and flexion contractures that can begin on the hands or feet and extend proximally. Cutaneous calcifications maybe noted on a plain film radiograph and confirmed on biopsy [19]. Lesions are frequently symmetrical, often located on the lower limbs, followed by the forearms. Idiopathic, rapid onset, unstable hypertension has been described prior to onset of skin lesions. Its systemic involvement of lungs, heart, diaphragm, liver or kidneys can vary. The international centre for research on NSF, led by Prof. Dr. S.E. Cowper, states that approximately 5 % are reported to have a fulminant course [20]. The Girardi Score (Fig. 1, Table 1) was proposed in 2011 based on reported clinical presentations and expert consensus, as no single laboratory test could be used as a gold standard to diagnose NSF. This encompassed identification of major and minor criteria on clinical findings, coupled with histological findings.

**Table 1:** Girardi score—definition and classification of clinical and histological findings [56]

Girardi score	
Clinical findings major criteria	Patterned plaques Joint contractures “Cobblestoning” Marked induration / Peaud’orange
Clinical findings minor criteria	Puckering / linear banding Superficial plaque / patch Dermal papules Scleral plaques (<45 years)
Histological findings	Increased dermal cellularity (Score +1) CD34+ cells with tram tracking (Score + 1) Thick & thin collagen bundles (Score + 1) Preserved elastic fibres (Score -1, if absent) Septal involvement (Score +1) Osseous metaplasia (Score +3)

### Pathophysiology

Recent theory suggests that administration of large amounts of GBCAs, (solely excreted via kidneys for earlier used formulations and again mainly excreted by kidneys in the remainder) persist in the body and may dissociate from their carrier ligands/chelates [16, 21, 22]. These may then bind with readily available phosphates, carbonates or citrates, and form insoluble molecules. Several authors mention histological findings of increased dermal collagen bundles, CD34+ fibroblast like cells, macrophages, mucin and transforming growth factor beta (TGFβ) in this cohort of patients [2, 4, 5, 16, 23–25]. Pre-existing renal disease has been the most prevalent patient characteristic.

Although the disassociated gadolinium theory has been the widely acknowledged trigger of NSF, questions of chelated gadolinium in combination with pre-existing cofactors have been raised [26]. Cofactors such as high dose erythropoietin treatment, pro-inflammatory state, high serum phosphate and calcium, and absence of ace inhibitor treatment, have also been linked to the appearance of NSF [27–30]. Chelated gadolinium such as gadodiamide and gadopentetate have been shown to directly stimulate macrophages and monocytes in vitro to release profibrotic cytokines and growth factors capable of initiating and supporting the characteristic tissue fibrosis [23, 31–33].

### Hepatic insufficiency

As evidence in published literature increased following the initial June 2006 alert, manufacturers of GBCAs were ordered by the FDA to add a “black box warning” the following year [34]. This elaborated on the alert to extend caution of GBCA use in any acute or chronic renal insufficiency patient (GFR < 30 mL/min/1.73 m2), or acute renal insufficiency of any severity due to the hepato-renal syndrome or in the perioperative liver transplantation period. A 2009 systematic review of NSF in liver disease patients found no compelling evidence to suggest liver disease in itself as being a risk factor for developing NSF. The authors concluded that NSF developed only in the setting of pre-existing severe renal insufficiency, irrespective of liver disease status [35].

### Pregnancy

To date, very little data is available regarding GBCA administration in pregnancy. Most of the published studies are either animal studies or patient cohorts that are understandably historic, and have small numbers and limited follow-up periods [36–42]. As evidence of gadolinium retention after exposure continues to pile and taking into consideration immature foetal renal function, recommendations are much more restrictive. Guidelines recommend that GBCAs should only be given to pregnant women when there is a very strong clinical indication. Breast feeding should be stopped for at least 24 hours. One of the more stable, macrocyclic gadolinium agents (gadoterate meglumine, gadoteridol, or gadobutrol) should be used in the lowest dose consistent with a diagnostic result [43].

### Excess chelate

The vast majority of GBCA preparations contain excess chelates to reduce or ensure absence of free gadolinium in the solution, and some studies have suggested the possibility of excess chelate to inhibit the collagenolytic properties of matrix metalloproteinase 1. The addition of excess chelate to non-ionic linear chelate dramatically reduces the acute toxicity [9, 37, 43]. Table 2 summates the commonly used GBCAs, their elimination pathway, reports of NSF and the amount of excess chelate within the preparations.

**Table 2:** Gadolinium based contrast agents—elimination pathway, last reported total number of administrations, occurrences of NSF and volume of excess chelate quantity [11]

Gadolinium based contrast agents	Elimination pathway	Number of reports		No. administrations (millions)	Excess chelate in preparation
		Unconfounded	Confounded		
Omniscan® (Gadodiamide)	Kidney	438	90	47	25 mmol/L
Optimark® (Gadoversetamide)	Kidney	7	11	0.8	35 mmol/L
Magnevist® (Gadopentetate dimeglumine)	Kidney	135	276	95	135 mmol/L
Multihance® (Gadobenate dimeglumine)	97 % Kidney 3 % Bile	0	8	6	1 mmol/L
Primovist® (Gadoxetic acid disodium salt)	50 % Bile 50 % Kidney	0	0	0.15	(Not known)
Vasovist® (Gadofosveset trisodium)	91 % Kidney 9 % Bile	0	0	0.05	(Not known)
Prohance® (Gadoteridol)	Kidney	1*	2	2.6	1 mmol/L
Gadovist® (Gadobutrol)	Kidney	1	13	12.3	2 mmo/L
Dotarem® (Gadoteric acid)	Kidney	1**	11	22.4	0

*Case published on 5 October 2009

**9 years prior to Dotarem administration, the patient had received an unknown GBCA. Case is still under investigation

## Treatment

Thus far, no consistently successful treatment for NSF has been proposed. Improving renal function slows or arrests NSF to allow for gradual reversal over time, and has been described in patients who received renal transplantation [44]. Dialysis helps to remove the contrast agent, but it cannot reverse the fibrotic tissue formation that has already occurred as a result of gadolinium deposition [26, 45]. With a full 4-hour dialysis session after administration, concentration levels comparison to predialysis have been shown to be to cleared to 88 % at 30 mins, 93 % at 90 mins, and 97 % respectively. After a third session, a 99.7 % clearance has been demonstrated [45]. Whether this would still be associated with development of NSF would require long-term follow-up of these patients. Other treatments such as oral and topical steroids have been tried with varying results [16, 46]. Extracorporeal photopheresishas shown good results in a small case series and in three patients who were also kidney/liver recipients [47–49]. Plasmapheresis was also utilized with acceptable results [50, 51]. Anecdotal evidence has been reported in the use of Cytoxan, thalidomide, ultraviolet therapy, physical therapy including deep massage technique, pentoxyfilline (at high doses), sodium thiosulphate, alefacept, and imatinib mesylate, and intravenous immunoglobulin (also at high dose and after renal transplantation) [17]. Not having mandatory reporting, the NSF Registry, led by Prof. Dr. S.E. Cowper, still mentioned over 380 cases in 2013 [20]. The highest incidence in Europe has been reported in Danish registry reports [52]. The true incidence of NSF may likely be under-reported, but in cases of confirmed NSF, renal transplantation should be made a priority.

## Guidelines

From 2006 onwards, international agencies such as the European Medicines Agency (EMA), the European Society for Urological Radiology (ESUR), the US Federal Drugs Agency (FDA), American College of Radiology (ACR) and the UK Royal College of Radiologists (RCR) have published alerts, precautions and recommendations on the use of GBCAs. Multiple publications, have since been gathered to form the body of evidence for NSF; however, the vast majority have been linked to the earlier types of contrast agents. The incidence of NSF has been reported to be on the decline after these recommendations were implemented.

While various different formulations are available on the market, not all have been associated with NSF. Linear GBCAs are considered the least stable, and have been linked to most cases of the development of NSF [3, 15]. These have often been linked to the background of an inflammatory process [16]. Macrocyclic GBCAs for MRI have also been developed.

A recent systematic review of MRI studies in the renal impaired noted that the majority of included studies were published prior to the FDA alert [53]. Half of the studies reported use of contrast types now mentioned by the EMA as having high incidence of NSF (Table 3).

**Table 3:** European Medicines Agency: categorisation of GBCAs according to NSF risk, based on their thermodynamic and kinetic properties [11]

High risk	
A. Linear non-ionic chelates B. Linear non-ionic chelates	A. gadoversetamide (OptiMARK®), gadodiamide (Omniscan®) B. gadopentetic acid (Magnevist®, Magnegita®, and Gado-MRT-ratiopharm*)
Medium risk Linear ionic chelates	Gadofosveset (Vasovist®), gadoxetic acid (Primovist®) and gadobenic acid (MultiHance®)
Low risk Macrocyclic chelates	Gadoteric acid (Dotarem®), gadoteridol (ProHance®) and gadobutrol (Gadovist®)

*Gadopentetic acid generics

In December 2007, the EMA recognized that the risk of developing NSF depends on the type of gadolinium-containing contrast agent used, and advised that these agents should be categorized into three groups. Following this categorization, if a GBCA is to be used in a high-risk patient, then the low risk category agents should be used. Risk and benefit analysis assessment and informed consent should be obtained. Always record the name and dose of the contrast agent used in the patient records. The use of high risk GBCAs in patients with acute kidney injury, end-stage renal disease or stage 4 and 5 chronic kidney disease is not recommended. Caution is advised in patients with stage 3 disease (eGFR between 30 and 59 ml/min/1.73 m^2^). A minimal 7-day interval should be observed between administrations [11, 12, 43].

## Conclusion

- Low risk gadolinium contrast agents as identified by the EMA should be the choice if CE MRI is to be carried out, but only after careful risk and benefit assessment. Informed consent should be obtained regarding GBCA administration. As appearance of NSF can occur from months to years after administration, clear documentation of date, dose and type of formulation used should be included in case notes.[13, 54].
- Dosage should be kept to a minimum, as higher doses have been linked to the development of NSF. A minimal 7-day interval should be observed between administrations [11, 14, 43, 46]. Post scan, a full 4-hour dialysis session should be arranged for dialysis-dependent patients [45]. Dialysis solely for contrast filtration is not recommended due to high risk of morbidity and mortality [11, 12].
- A pre-existing pro inflammatory state in the renal impaired is a high risk factor [2, 3, 16, 17, 55].
- Liver insufficiency in itself is not a contraindication; however, patients may also have coexisting renal insufficiency and thus carry a risk of NSF [35].
- There is insufficient reported data regarding GBCA use in the pregnant and neonate population [40–42].
- Studies exploring efficacy of stronger magnetic fields, non-contrast or low dosage, and diagnostic test accuracy studies would aide in clinical decision making. Continuing follow-up and research will be needed on low-risk formulations in the long term.
